# Outcome of a rabbit model for late irradiation effects in mandibular oral mucosa and bone: A pilot study

**Published:** 2020-12-11

**Authors:** R. Helmers, D. M. J. Milstein, N. F. Straat, H. M. Rodermond, N. A. P. Franken, C. D. Savci-Heijink, H. H. de Boer, J. de Lange

**Affiliations:** ^1^Department of Oral and Maxillofacial Surgery, Amsterdam University Medical Centre (UMC), Location: AMC, University of Amsterdam, Meibergdreef 9, 1105 AZ, Amsterdam, The Netherlands,; ^2^Academic Centre for Dentistry Amsterdam, University of Amsterdam and VU University, Gustav Mahlerlaan 3004, 1081 LA, Amsterdam, The Netherlands,; ^3^Laboratory of Experimental Oncology and Radiobiology, Amsterdam UMC, Location: AMC, University of Amsterdam, Meibergdreef 9, 1105 AZ, Amsterdam, The Netherlands,; ^4^Department of Radiation Oncology, Amsterdam UMC, Location: AMC, University of Amsterdam, Meibergdreef 9, 1105AZ Amsterdam, The Netherlands,; ^5^Department of Pathology, Amsterdam UMC, Location: AMC, University of Amsterdam, Meibergdreef 9, 1105 AZ, Amsterdam, The Netherlands

**Keywords:** angiopathology, experimental model, late irradiation injury, microcirculation, sidestream dark-field imaging, radiotherapy

## Abstract

**Background/Aim/Objective:**

Late side effects of radiotherapy (RT) in the treatment for head and neck (HN) malignancies involve an inadequate healing response of the distressed tissue due to RT-induced hypovascularity. The aim of this study was to develop a pilot model in which vascular alterations associated with the onset of late irradiation (IR) injury could be measured in rabbit oral mucosa and mandibular bone.

**Materials and Methods:**

Eight male New Zealand white rabbits were divided over four treatment groups. Group I-III received four fractions of RT (5.6 Gy, 6.5 Gy, and 8 Gy, respectively) and Group IV received 1 fraction of 30 Gy. Oral microcirculatory measurements were performed at baseline (before RT) and once a week during 11 consecutive weeks after RT assessing perfusion parameters, that is, total vessel density (TVD), perfused vessel density (PVD), proportion of perfused vessels (PPV), and microvascular flow index (MFI). Post-mortem histopathology specimens were analyzed.

**Results:**

Five weeks after RT, TVD, and PVD in all groups showed a decrease of >10% compared to baseline, a significant difference was observed for Groups I, II, and IV (*P*<0.05). At T11, no lasting effect of decreased vessel density was observed. PPV and MFI remained unaltered at all-time points. Group IV showed a marked difference in scattered telangiectasia such as microangiopathies, histological necrosis, and loss of vasculature.

**Conclusion:**

No significant lasting effect in mucosal microcirculation density due to IR damage was detected. Observed changes in microcirculation vasculature and histology may align preliminary tissue transition towards clinical pathology in a very early state associated with late IR injury in the oral compartment.

**Relevance for Patients:**

Enhancing knowledge on the onset of late vascular IR injury in the HN region could help the development, monitoring, and timing of therapies that act on prevention, discontinuation, or repair of radiation pathology.

## 1. Introduction

Radiotherapy (RT) has an essential role in the treatment of head and neck (HN) cancer patients. Irradiation (IR) of HN malignancies concomitantly elicits acute and late injuries to healthy tissues resulting in clinical side effects such as oral mucositis, dry mouth, ulceration, and osteoradionecrosis (ORN) [1,2]. Acute side effects of RT usually heal by the end of treatment, whereas possible late effects due to reduced vascularity and fibrosis tend to occur after a period of several months to years after treatment [3-5]. RT-induced hypovascularity could progress in a hypocellular and hypoxic tissue state in which the distressed tissue is unable to establish an adequate healing response to injury and eventually succumbs to tissue breakdown and ORN [6]. ORN in HN has a reported incidence of 2-22% [7-9] and is a potentially mutilating and complicated pathology to manage.

Experimental research fulfills an essential role in providing standardization of a model and controlled environments for studying pathophysiology and interventional responses associated with HN IR injury. In clinical studies, standardization in design is difficult to achieve as tumor location and radiation dose vary between patients. Late IR tissue histopathology involving human jawbone presents as a progressive state of hypovascularity and fibrosis [10,11]. Experimental studies that aimed to create a late IR tissue injury model in the HN region were previously conducted. Distraction osteogenesis on rabbit irradiated mandibles showed a general reduction in vascularity, osteoblastic activity, and bone regeneration compared to non-IR controls [12-15]. Increased fibrosis and decreased vascularity in the soft tissues surrounding the irradiated mandible was observed after 5 fractions of 15 Gy in a rat IR model [4]. In another model using mini-pigs, an increased amount of fibrosis and decreased vascular lumens in the mandible was proportionally dependent with the dose administered (25-70 Gy) 14-24 weeks after RT [16]. Studies directed at evaluating the effects of RT mainly investigated tissue quality at functional or subcellular level by measurements obtained using X-ray microtomography (micro-computed tomography) [15,17,18], microangiography [19], transmission electron microscopy [4], and by looking at histopathology [4,10,12-20]. A disadvantage associated with most of the techniques listed above is that they are invasive and often require tissue manipulation and/or injecting a contrast medium for data acquisition. To further advance knowledge on oral tissue microvascular integrity and its perfusion in response to IR, the oral microcirculation was measured *in vivo* using a noninvasive, optical spectroscopic-based handheld imaging system, and sidestream dark-field imaging (SDFI). SDFI has extensively been used at the patient bedside and experimental setting to monitor and assess pathophysiological states associated with shock (e.g., cardiogenic, hypovolemic, and septic) [21,22], interventional endpoints and therapeutic responses associated with microvascular alterations in critical care patients, wound healing, and the side effects of cancer chemotherapy and RT [23-25]. Decreased capillary density, irregular dilatation of blood vessels, and telangiectasia were associated with late IR effects in human oral mucosa [24]. In the present study, parameters coinciding with microvascular blood flow dynamics and microvascular density were examined and compared between baseline and then after IR.

The aim of this study was to develop a pilot translational IR model in which microvascular alterations associated with the onset of late IR injury could be measured in rabbit oral mucosa and mandibular bone. Microcirculatory changes in vessel density and blood flow were quantified and compared between baseline and after IR. To ascertain the translational accuracy of a late IR injury model, we test the hypothesis that cumulative IR dosages equal to 22.4 Gy, 26 Gy, 30 Gy, and 32 Gy would result in oral mucosal and mandibular bone histological changes similar to humans.

## 2. Materials and Methods

The institutional Animal Experimentation Committee of the Academic Medical Center of the University of Amsterdam approved the protocols and guidelines for this study (Ref. No. DFL103187). The care and use of animals were conducted in accordance with the EU Directive 2010/63/EU (September 22, 2010) and the Dutch Act on Laboratory Animal Experiments.

### 2.1. Animals

Eight male specific-pathogen-free New Zealand white rabbits (*Oryctolagus cuniculus*) (Charles River Laboratories France, L’Arbresle Cedex, France) were randomly divided between four groups. Each group received a different total IR dose. Mean body weight at start of the study was 2.72±0.16 kg; weighing was carried out weekly throughout the entire period of the study. Animals were housed separately in large cages (R-SUITE Enriched Rabbit Housing, Techniplast SpA, Buguggiate [Varese], Italy) in a 24-h light-controlled room (12 h light/dark cycle) that was maintained at a constant temperature of 22±1°C and humidity of 55±10%. Consumption of standard food pellet diet (LK-04, AB Diets, Woerden, The Netherlands) and water (acidified to pH 2.7) was available *ad libitum*. Sedation to perform microcirculation measurements, blood sampling, and administration of RT was achieved though administration of subcutaneous injection of a mixture of ketamine (Nimatek, Eurovet Animal Health BV, Bladel, The Netherlands; 15 mg·kg^−1^) and dexmedetomidine (Dexdomitor, Pfizer Animal Health BV, Capelle aan den Ijssel, The Netherlands; 0.2 mg·kg^−1^). To monitor general hematological health, a blood sample was obtained at every measurement time point by drawing 1 mL of blood from the central ear artery, anticoagulated in K2E 7.2 mg ethylenediaminetetraacetic acid (EDTA) BD Vacutainer® 4 mL tubes (Becton, Dickinson & Co., Plymouth, United Kingdom) and analyzed by an automated Sysmex XE-5000 (Sysmex Corporation, Kobe, Japan). Hereafter, microcirculation measurements and RT were performed. After all data collection and RT atipamezole (Antisedan^®^, Pfizer Animal Health BV, Capelle aan den IJssel, The Netherlands; 1 mg·kg^−1^ sc) was administered to reverse anesthesia. At the end of the study, the animals were sacrificed using sodium pentobarbital (Euthasol^®^, AST Farma BV, Oudewater, The Netherlands; 120 mg·kg^−1^ iv) administered through the lateral ear vein. After sacrifice, the mandibles with overlaying mucosa were dissected and excised for post-mortem histopathological analysis. All animals were exposed to the same experimental proceedings: Weighing, sedation, baseline, and consecutive weekly whole blood counts, microcirculation measurements, RT, reversal of anesthesia, sacrifice, and mandibular excision.

### 2.2. IR

IR was performed using an Xstrahl X-ray generator (Xstrahl Ltd., Surrey, United Kingdom), operated at 225 kV and 15 mA and inherent filtration with 1 mm Cu, a square tube collimator with an area of 8×8 cm^2^. After being anesthetized, the fur on the right lower jaw was shaved, the right side of the mandible was marked and subsequently the animals were transported from their housing facility to the IR lab at the department of experimental RT. Upon arrival at the experimental RT lab, each rabbit was placed in a dorsally recumbent position on a table-top under the X-ray generator. The mandible faced the focus source of the IR with a beam to subject distance of 20 cm. A lead plate with a rectangular recess of 2×6 cm was positioned on a foam scaffold in between the IR source and the mandible. A smaller lead shield to protect the eyes, underlying intraoral tissues and maxillary region was inserted intraorally to isolate the mandible for IR. The focus was oriented to unilateral IR to the right side of the mandible and the left side was used as a non-IR control. All animals were closely monitored during the treatment procedure after which they were transported back to their housing area where reversal of anesthesia was administered with 1 mg·kg^−1^ sc atipamezole.

### 2.3. IR protocol

All study animals were randomly divided into four groups (I, II, III, and IV) of two animals each and each group received different total IR doses (i.e., 22.4 Gy, 26 Gy, 32 Gy, and 30 Gy, respectively). Groups I, II, and III received four fractions (5.6 Gy, 6.5 Gy, and 8 Gy, respectively, with a dose rate of 110 cGy/min) in 2 consecutive weeks on day 0, 3, 6, and 9. Group IV received the total dose (30 Gy with a dose rate of 110 cGy/min) in 1 fraction on day 0. All IR protocol procedures were performed by the same investigators (RH, HMR, and NAPF).

### 2.4. Microvascular imaging technique

The microcirculation in the oral mucosa covering the lingual aspect of the mandible was examined and measured using a commercially available SDFI instrument, also known as MicroScan (MicroScan Video Microscope System, MicroVision Medical, Amsterdam, The Netherlands). Details on SDFI are described elsewhere [26,27]. In brief, stroboscopic emission of 530-nm wavelength (green light) through LEDs placed at the tip of the lens probe epi-illuminate the tissue and partially gets scattered and absorbed by hemoglobin (Hb) in red blood cells (RBCs). The difference in absorption and scattering produces clear images of dark circulating RBCs in the vascular lumen contrasted by a light background. Video clips were captured through a 5× objective lens system (equal to 380× onscreen magnification) on a charge-coupled device video camera with 720×576-pixel resolution. The imaged tissue segments were 1.0×0.75 mm^2^. DVI tapes on a Sony DSR-11 DVCAM™ recorder (Sony, Shinagawa-ku, Tokyo, Japan) were used for storage and were viewed on a 19-inch Samsung SyncMaster 932mv LCD monitor (Samsung, Seoul, South Korea) with a 1440×900-screen resolution.

### 2.5. SDFI measurements

In all groups, microcirculatory measurements were performed at baseline (before RT) and every week for 11 consecutive weeks. All measurements were performed by the same investigator (RH) in the same examination room, kept at a constant temperature of 22±1°C. After inducing anesthesia and blood sampling, each animal was placed in a prone position on a flat surface with a cotton dental roll wedged between the maxillary and mandibular (pre-) molars to keep the mouth open and providing space for placement of the imaging probe. Moisturizing of the mouth for good contact between mucosa and probe tip was performed by irrigation of the oral mucosa with a warm (37°C) sterile physiological saline solution (0.9% NaCl). The imaging probe was covered with a sterile disposable cap (MicroScan Lens, MicroVision Medical, Amsterdam, The Netherlands) and placed gently perpendicular on the lingual aspect of the mandibular mucosa on the diastema (gap) between the incisors and the premolars and aiming for full contact without applying any pressure thereby avoiding vascular occlusion (pressure) artifacts. Microcirculation recordings were acquired while withdrawing and advancing the lens probe from release and contacting the tissue surface to record segments free of pressure artifacts. A 2-min video clip was obtained of five adjacent sites on the IR side and the contralateral non-IR side of the inside of the mandible in each subject at each time point.

### 2.6. Microcirculation data analysis

The DVI tapes used for storage of the microcirculation data were converted to digital AVI files with Adobe Premier Pro 1.5 (Adobe Systems Incorporated, San Jose, California, USA) and then analyzed offline. After separating the sequentially recorded sites per time point, 1 clip of each site (4 in total) was selected based on quality judged by good image brightness/contrast, sharpness, clarity, and absence of pressure artifacts in conformity with guidelines established by a round table conference based on microcirculation data acquisition and analysis consensus meeting [28]. Hereafter, all microcirculation data were analyzed at random by two investigators (RH, NFS) using the Automated Vascular Analysis software package (AVA v3.02, MicroScan Video Microscope System, MicroVisionMedical, Amsterdam, The Netherlands). Analysis of microcirculatory data was performed on vessels with diameters <25 mm for total vessel density (TVD; mm vessel/mm^2^), perfused vessel density (PVD; mm perfused vessel/mm^2^), proportion of perfused vessels (PPV; %) [28], and microvascular flow index (MFI). MFI was determined by describing the type of flow in each quadrant of the clip using the following scoring system: Absent (0), intermittent (1), sluggish (2), or normal (3) [29,30].

### 2.7. Mandibular histopathology

Following sacrifice, the mandible of each subject was carefully dissected and fixed using 4% buffered formaldehyde. Mandibular bone cross-sections were prepared as 4 mm thick slices, which were demineralized in EDTA and processed into standard, paraffin embedded, 8 mm thick histological slides. All samples were (immuno-) histochemically stained with hematoxylin and eosin, Elastica van Gieson and analyzed under a light microscope (BX50, Olympus, Tokyo, Japan) at ×100 magnification. The samples were analyzed for general histological parameters for tissue integrity and reactive changes such as hemorrhage, acute and chronic inflammation, osteoblast and osteoclast activity, loss of vascular structures, fibrosis, and necrosis. Current study samples were compared to the histology of two healthy age-matched rabbit mandibular material obtained from a tissue bank of a former study (Ref. No. DFL101932). All histological analyses were performed by the same investigator (HHdB).

### 2.8. Statistical analysis

As no standardized experimental IR rabbit model was available in literature, this pilot study aimed to determine which dose could induce measurable late IR damage in tissue. Since research data on the effects of late IR effects on *in vivo* microcirculation was not available, this study served as a basis for orientation and design. We aimed to detect an estimated difference between baseline and post-IR in TVD of approximately 8-10% in the proposed study groups. A minimum of two animals per group was used to achieve procedural endorsement. As all study groups consist of just two rabbits, the Friedman test, a non-parametric test with no correction for multiple comparisons, was applied to all groups to detect a statistical difference (*P*<0.05) over time compared to baseline. The data of all parameters were separately analyzed (TVD, PVD, PPV, MFI, body weight, and whole blood counts) and presented in mean±SD. Mean TVD and PVD measurements were converted into percentages and to correct for innate biological variations, datasets were normalized with respect to baseline. Data analysis was performed with Prism 8.3.1 for macOS (GraphPad Software, LLC, San Diego, California, USA).

## 3. Results

All animals responded well to the IR model and the repeated microcirculation measurement protocol with no signs of distress or discomfort. Transportation of the anesthetized animals to and from the IR lab was uneventful. After returning the animals to their housing and reversing anesthesia, no signs of distress or distressed recovery were observed. At T11 groups, I-III showed significant increases in body mass compared to baseline (*P*<0.05). When body mass was examined separately in each animal, a marked decrease in body mass was noted in one animal from Group IV, in which weight loss started from T7 and resulted in a 16% lower body mass at T11 compared to T6. Whole blood count parameters (Hb, platelet [PLT], RBC, white blood cell [WBC]) remained within normal reference value range [31,32] throughout all time points. Groups I, III, and IV showed a recurrent significant decrease in PLT count and Group IV a significant decrease in WBC count at different time points. Whole blood count parameters and body mass are presented in Table 1.

**Table 1:** Summary of body weight and whole blood counts for all animals at each time point.

	BL (T0)	Day 7 (T1)	Day 14 (T2)	Day 21 (T3)	Day 28 (T4)	Day 35 (T5)
Mass (kg)						
G1	2.75±0.13	2.35±0.43	2.96±0.37	3.09±0.30	3.22±0.39	3.26±0.39
G2	2.73±0.43	2.80±0.25	3.02±0.34	3.14±0.35	3.75±0.29	3.33±0.45
G3	2.64±0.14	2.87±0.06	3.03±0.04	3.11±0.02	3.11±0.02	3.19±0.01
G4	2.77±0.06	2.80±0.01	2.88±0.04	3.04±0.07	3.19±0.06	3.29±0.02
Hb (mmol/L)						
G1	8.1±0.5	8.4±0.4	8.0±0.6	8.1±0.5	8.1±0.2	8.4±0.5
G2	8.2±0.5	8.2±0.2	7.8±0.1	8.0±0.0	8.3±0.1	8.4±0.0
G3	8.0±0.6	8.2±0.1	7.6±0.3	7.5±0.1	7.6±0.1	7.8±0.1
G4	8.4±0.4	8.5±0.3	8.3±0.4	7.9±0.4	8.1±0.2	8.2±0.0
Ht (L/L)						
G1	NA±NA	0.42±0.02	0.40±0.03	0.40±0.02	0.40±0.00	0.42±0.02
G2	NA±NA	0.41±0.01	0.39±0.01	0.40±0.00	0.41±0.00	0.42±0.00
G3	NA±NA	0.41±0.02	0.39±0.02	0.39±0.01	0.39±0.01	0.40±0.01
G4	NA±NA	0.42±0.03	0.41±0.02	0.39±0.02	0.41±0.02	0.42±0.00
RBC (×10^12^/L)						
G1	6.42±0.21	6.57±0.16	6.25±0.40	6.30±0.37	6.27±0.19	6.45±0.35
G2	6.23±0.27	6.31±0.21	5.96±0.01	6.07±0.07	6.22±0.01	6.35±0.01
G3	6.43±0.74	6.62±0.46	6.12±0.57	6.01±0.24	6.00±0.28	6.06±0.42
G4	6.86±0.02	6.94±0.15	6.67±0.17	6.44±0.05	6.41±0.13	6.61±0.40
WBC (×10^9^/L)						
G1	7.85±0.86	7.93±0.07	6.42±0.78	7.91±0.54	6.79±0.30	7.40±0.16
G2	8.18±1.57	8.71±1.61	7.16±0.59	6.52±0.62	8.52±1.43	8.12±0.64
G3	7.92±1.16	8.03±0.30	6.27±0.02	6.95±1.07	7.76±2.61	7.25±2.04
G4	8.65±0.44	7.78±1.17	7.50±0.59	7.43±1.56	8.25±1.83	7.86±0.53
PLT (×10^9^/L)						
G1	260±25	251±34	234±17	193±28	137±34**	155±3**
G2	294±18	356±10	322±28	216±13	214±23	225±37
G3	192±40	230±34	164±52	145±40	174±75	139±37*
G4	386±52	400±64	336±39	361±74	322±26	283±51*
Mass (kg)						
G1	3.37±0.41	3.42±0.48	3.51±0.49	3.59±0.48	3.63±0.53	3.67±0.53
G2	3.42±0.49	3.51±0.51	3.57±0.49	3.65±0.49	3.68±0.59	3.70±0.61
G3	3.27±0.01	3.23±0.11	3.25±0.15	3.25±0.16	3.27±0.15	3.32±0.05
G4	3.39±0.04	3.34±0.18	3.33±0.33	3.34±0.45	3.38±0.56	3.17±0.44
Hb (mmol/L)						
G1	8.2±0.0	8.5±0.1	8.6±0.1	8.4±0.1	NA±NA	8.6±0.4
G2	8.4±0.1	8.6±0.1	8.7±0.1	8.5±0.1	NA±NA	8.8±0.1
G3	8.0±0.2	8.3±0.1	8.2±0.4	8.2±0.0	NA±NA	8.4±0.5
G4	8.6±0.1	8.7±0.1	9.3±0.5	9.0±0.3	9.2±0.3	9.2±0.3
Ht (L/L)						
G1	0.40±0.01	0.42±0.00	0.42±0.01	0.44±0.00	NA±NA	0.44±0.02
G2	0.41±0.00	0.42±0.00	0.42±0.00	0.43±0.01	NA±NA	0.45±0.00
G3	0.39±0.01	0.41±0.01	0.41±0.01	0.43±0.01	NA±NA	0.43±0.03
G4	0.42±0.01	0.43±0.01	0.45±0.03	0.46±0.01	NA±NA	0.47±0.00
RBC (×10^12^/L)						
G1	6.46±0.14	6.61±0.10	6.66±0.04	6.54±0.11	6.75±0.13	6.64±0.16
G2	6.36±0.03	6.46±0.04	6.54±0.08	6.40±0.08	6.55±0.20	6.67±0.06
G3	6.18±0.41	6.43±0.17	6.41±0.04	6.44±0.33	6.31±0.49	6.52±0.54
G4	6.81±0.28	6.83±0.57	7.24±0.85	7.11±0.68	7.18±0.41	7.28±0.28
WBC (×10^9^/L)						
G1	7.53±1.27	7.63±0.65	7.53±0.50	7.21±0.74	NA±NA	7.24±0.74
G2	8.36±1.82	6.89±0.80	7.51±0.16	6.01±1.18	NA±NA	8.57±0.06
G3	7.02±0.32	7.88±1.34	7.32±2.27	8.32±1.15	NA±NA	9.88±2.89
G4	6.82±0.93	6.54±1.75*	8.16±0.39	8.41±0.03	NA±NA	8.52±0.43
PLT (×10^9^/L)						
G1	221±60	225±65	192±3	201±5	242±4	211±11
G2	304±41	242±6	208±100	174±142	294±30	258±50
G3	192±14	184±30	163±23	155±37	191±13	203±4
G4	410±96*	255±144*	325±127	298±116	377±43	321±139

All data are presented in means±SD. BL: Baseline; Hb: Hemoglobin; Ht: Hematocrit; RBC: Red blood cells; WBC: White blood cells; PLT: Platelets; NA: Not available.

** *P*<0.01 versus T0, Friedman test,

* *P*<0.05 versus T0, Friedman test

### 3.1. IR-related clinical observations

**Figure 1:**
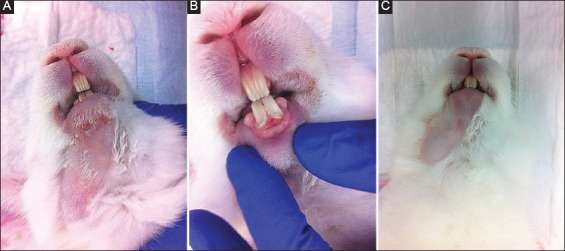
Photographs illustrating Group IV subjects (single dose of 30 Gy) with erythema of the irradiated skin (A) and signs of mucositis around the area of the lower incisors (B) at T2 and the absence of extraoral mandibular fur (C) in the irradiated region at T5.

In Group I, II, and III, no remarkable clinical observations were noted. At T2 both animals in Group IV showed erythema of the irradiated skin (Figure 1A) and one subject showed signs of mucositis around the area of the lower incisors (Figure 1B). These observations subsided by T3. Furthermore, both animals in Group IV showed no regrowth of fur at the site of IR after 5 weeks (T5) (Figure 1C), a notable difference compared to the other groups in which all subjects showed full fur regrowth by this time point (T5). At T8 general fur, regrowth started to appear in Group IV and was fully recovered by T11.

### 3.2. Mandibular mucosa microcirculation response to IR

Good image quality (i.e., brightness/contrast, sharpness, clarity, and absence of pressure artifacts) was obtained with the MicroScan on the lingual aspect of the mandibular mucosa. In each animal (*n*=8), four clips in total were obtained per time point; a total of 384 clips were analyzed (12-time points).

**Figure 2:**
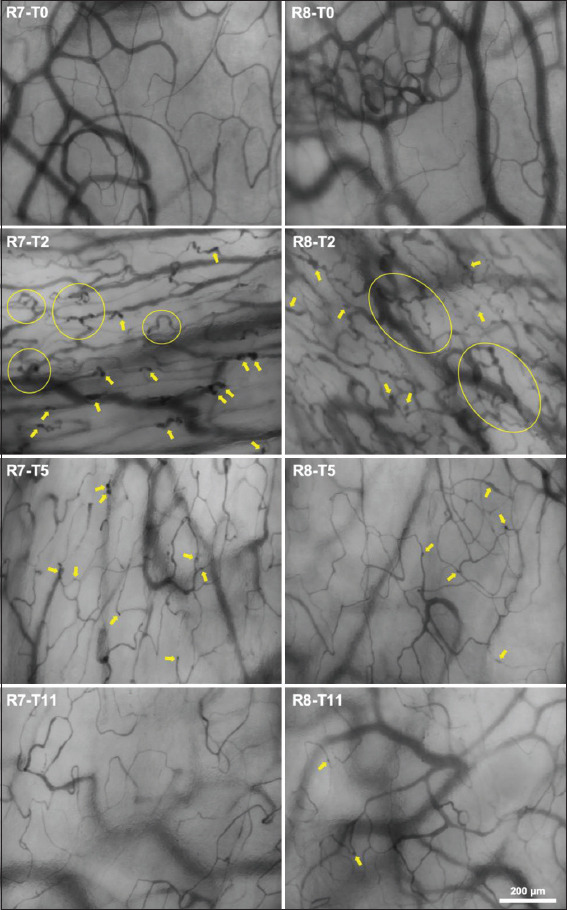
A series of MicroScan microcirculation image frames obtained from rabbit 7 and 8 (R7, R8; Group IV) at the lingual aspect of the mandibular mucosa. Frames at baseline (T0) and 2 weeks (T2), 5 weeks (T5), and 11 weeks (T11) after irradiation (IR) are presented. Yellow arrows and encircled areas point to the microangiopathy phenomenon observed in the form of bud-like telangiectasias. Total vessel density (TVD) was reduced in T11 compared to T0 (P<0.05).

Table 2 presents a summary of the analyzed microvascular parameters. Baseline means TVD and PVD for all groups was 26±3 mm/mm^2^. At T5, TVD and PVD in all groups showed a decrease of >10% compared to baseline, a significant difference was only observed for Groups I, II, and IV (*P*<0.05) and return to baseline TVD and PVD was first observed at T8 in Group III. In addition, in Group IV, marked changes in the microcirculation presenting as multiple bud-like telangiectasias were observed at all-time points with the most intense presentation at T2 in both animals (Figure 2). PPV and MFI remained unaltered across all time points.

**Table 2:** Summary of microcirculation data (vessels with diameter <100 µm).

	BL (T0)	Day 7 (T1)	Day 14 (T2)	Day 21 (T3)	Day 28 (T4)	Day 35 (T5)
TVD (mm/mm^2^)						
G1	28±5	25±1	29±1	25±2	27±2	21±1**
G2	25±1	25±3	26±1	28±3	27±1	20±1**
G3	27±1	31±0**	27±1	30±2	26±4	23±2
G4	24±1	29±4	22±4*	24±0	21±1*	21±2*
TVD (%)						
G1	100±0	91±21	107±17	92±9	99±25	78±16
G2	100±0	101±9	102±1	110±5	106±3	81±1
G3	100±0	115±3	99±2	109±12	96±12	83±10
G4	100±0	120±11	90±18	100±3	88±9	88±11
PVD (mm/mm^2^)						
G1	28±5	25±1	29±1	25±2	27±2	21±1**
G2	25±1	25±3	26±1	28±3	26±1	20±1**
G3	27±1	31±0**	27±1	30±2	26±4	23±2
G4	24±1	29±4	22±4*	24±0	21±1*	21±2*
PVD (%)						
G1	100±0	91±21	107±17	92±9	99±25	78±16
G2	100±0	101±9	102±1	110±5	105±2	81±1
G3	100±0	115±3	99±2	109±12	96±12	83±10
G4	100±0	120±11	90±18	100±3	88±9	88±11
PPV (%)						
G1	100±0	100±0	100±0	100±0	100±0	100±0
G2	100±0	100±0	100±0	100±0	100±1	100±0
G3	100±0	100±0	100±0	100±0	100±0	100±0
G4	100±0	100±0	100±0	100±0	100±0	100±0
MFI (0,1,2,3)						
G1	3	3	3	3	3	3
G2	3	3	3	3	3	3
G3	3	3	3	3	3	3
G4	3	3	3	3	3	3
TVD (mm/mm^2^)						
G1	20±2**	21±0**	26±3	25±1	25±2	25±1
G2	20±2**	24±2	24±0	23±0**	24±3	26±1
G3	26±2	25±0	27±0	26±3	30±5*	28±2
G4	22±0**	24±1	23±4*	25±2	26±4	23±1*
TVD (%)						
G1	74±19**	78±15**	94±8	90±13	91±25	91±21
G2	81±3	97±2	97±3	92±5	94±15	105±10
G3	95±2	93±2	101±6	97±6	110±13	101±4
G4	90±3	97±8	94±19	105±11	106±21	96±6
PVD (mm/mm^2^)						
G1	20±2**	21±0**	26±3	25±1	25±2	25±1
G2	20±2**	24±2	24±0	23±0**	24±3	26±1
G3	26±2	25±0	27±0	26±3	30±5*	28±2
G4	22±0**	24±1	23±4*	25±2	26±4	23±1*
PVD (%)						
G1	74±19**	78±15**	94±8	90±13	91±25	91±21
G2	81±3	97±2	97±3	92±5	94±15	105±10
G3	95±2	93±2	101±6	97±6	110±13	101±4
G4	90±3	97±8	94±19	105±11	106±21	96±6
PPV (%)						
G1	100±0	100±0	100±0	100±0	100±0	100±0
G2	100±0	100±0	100±0	100±0	100±0	100±0
G3	100±0	100±0	100±0	100±0	100±0	100±0
G4	100±0	100±0	100±0	100±0	100±0	100±0
MFI (0,1,2,3)						
G1	3	3	3	3	3	3
G2	3	3	3	3	3	3
G3	3	3	3	3	3	3
G4	3	3	3	3	3	3

All data are presented in means±SD. BL: Baseline; MFI: Microvascular flow index;PPV: Proportion of perfused vessels; PVD: Perfused vessel density; TVD: Total vessel density.

** *P* <0.01 vs. T0, Friedman test.

* *P*<0.05 vs. T0, Friedman test

### 3.3. Mandibular bone histology

**Figure 3:**
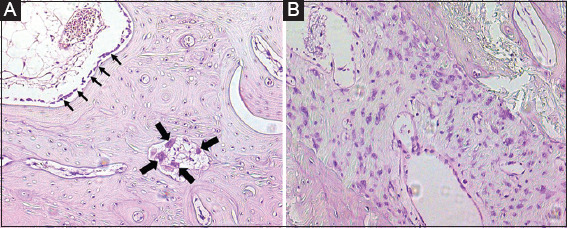
Micrographs of the bone histology samples stained with hematoxylin and eosin. Panel A shows plump and enlarged osteoblasts (small arrows) and an increased number of multinucleated osteoclasts (large arrows), signaling increased bone turnover. Panel B shows enlarged atypical nuclei of the fibroblasts in the fibrous tissue, commonly associated with radiation-induced changes.

Mandibular histological specimens from Groups I-IV were compared to healthy age-matched specimens. Group I did not show any deviating findings compared to healthy non-irradiated tissue. There was a gradual increase of pathological changes observed in Groups II-IV. Group II showed a slight increase of congestion of the vascular structures in bone and soft tissue with focal hemorrhage/extravasation of erythrocytes in the surrounding soft tissue. Furthermore, a slight increase in osteoblastic and osteoclastic activity was noted. The teeth showed no pathological changes except for a focal irregularity of cement which was anatomically present on the lingual site. Group III showed a marked loss of vascularity of the bone and soft tissue, with an increase of fibrotic soft tissue. The bone tissue was vital, although marked osteoblastic activation and an increase of osteoclastic bone resorption were observed (Figure 3A). The teeth had an avascular pulp with irregular deposits of cement at the interface between the teeth and the adjacent soft tissue on the lingual side. Group IV showed further general depletion of vascular structures and an increase of fibrosis in bone and soft tissue when compared to Group III. The fibroblasts showed radiation-induced changes (i.e., enlarged, vesicular nuclei) (Figure 3B). The bone tissue showed small necrotic fields, although the majority of the bone tissue was vital. Some of the vital bone was devoid of an osteoblastic lining (periosteum), while multifocally, an increase in resorptive activity by multinucleated osteoclasts was observed. The teeth had an avascular pulp and showed partially resorbed cement on the lingual side of the teeth.

None of the groups showed thrombosis, vasculitis, or reactive changes of the endothelium. Finally, no evident difference in observations between the irradiated site and the contralateral site was observed.

## 4. Discussion

The aim of the present study was to develop a pilot IR model in which the effects and onset of late IR injury could be established and measured noninvasively in the oral mucosal microcirculation and in mandibular bone by histological analysis. Interestingly, at the end of 11 weeks, the results showed that RT administered in different fractions and intensities corresponding with cumulative doses equivalent to 22.4 Gy, 26 Gy, 30 Gy, and 32 Gy established no evident altered perfusion or lasting microvascular changes besides bud-like telangiectasia observed after a single dose of 30 Gy. There was a gradual IR dose-dependent increase in pathophysiological changes observed in Groups II-IV that presented most prominently as reduced vascularity, fibrosis of soft tissue and bone, and necrosis of teeth and bone tissue after a single dose of 30 Gy.

SDFI previously showed changes in oral microcirculation in rabbit wound vascularization studies in response to chemotherapy and hyperbaric oxygen therapy [23,25]. Although no clear late IR effect could be detected in our microcirculation data at T11 in all groups, there was a notable recurring effect around T5 regarding clinical side effects (no regrowth of hair at side of IR in Group IV), reduced TVD and PVD (Group I, II and IV), and decreased PLTs (Group I, III and IV) compared to baseline. This suggests that our study may reflect microcirculation dynamics potentially associated with the onset of IR injury in a transition phase before late IR injury. The observation of a transient decrease in functionality of the microcirculation could be a result of leaking or thrombosed capillaries injured by IR. A decrease in PLTs associated with locoregional IR, which is supported although not valued as clinically relevant by the scarcely available literature [33,34], could further reduce angiogenesis and repair in compromised IR tissue as they contain PLT-derived growth factors [35]. Recently, in an observational clinical study, late IR injury in oral mucosal microcirculation was described as showing significant rarefaction in capillary density, irregularly enlarged blood vessel diameters, and telangiectasias [24]. Interestingly, Group IV showed scattered bud-like telangiectasia in the microcirculation, with this appearing most prominently at T2, that is, 2 weeks after RT. Since none of the other groups showed this type of phenomenon in the microcirculation, a higher IR fraction dose might be necessary to induce comparable vascular reactions associated with RT in humans in the present animal model.

The histology of mandibular bone of Groups III and IV showed an increase in fibrotic interstitial tissue and loss of vascular structures in bone and soft tissue compared to the histology of healthy rabbit mandibular bone. Increased fibrosis in IR tissue was in agreement with other study reports [10,16,18,19,36] and was described as a decrease in vessel lumen space and an increase in the thickness of the tunica intima [10,16]. Group IV histologically showed small fields of bone necrosis marked by empty osteocyte lacunae 11 weeks after a single fraction of 30 Gy. Interestingly, another study reported a significant decrease in bone formation rate and vascular lumen diameter and an increase in tunica intima thickness starting from 14 weeks after IR (highest total dose of 23.6 Gy, total follow-up 26 weeks) measured by dynamic histomorphometry and histology assessment [16]. In their study, bone necrosis only followed after surgical intervention post-IR, which might indicate a less severe impact of IR-dose alone when compared to our findings after a single fraction of 30 Gy. A significant decrease in osteocytes and increase in empty lacunae (osteocyte death) in the bone of irradiated rabbit mandibles was correspondingly demonstrated in another study after administration of a total dose varying between 35 Gy and 45 Gy (in 5 fractions) [18]. In addition, an increase of myofibroblasts invading osteocytes and surrounding sequesters was noted, corresponding with the known chronic fibrotic state associated with RT [18,36]. Finally, Group IV showed a lack of osteoblast lining (periosteum), increased resorption by osteoclast activity, and decreased vascularization, which is in line with observations describing human ORN mandibles [10]. The absence of periosteum might be the first step preceding soft tissue ulceration that eventually succumbs to bone necrosis and exposure. However, bone necrosis and exposure were not observed in our model.

Previous rabbit models studying IR effects in the HN region applied different treatment approaches such as total radiation dose, duration, and number of fractions administered and technique of IR (X-ray tube, linear accelerator, and cobalt therapy) [12-15,17-20,37-39]. After dosimetry calculation and comparisons with previous investigations, a cumulative dose ranging between 22.4 to 32 Gy was chosen for this pilot study with the purpose to select a clinically relevant spectrum of IR therapy approaches. Since cell and tissue turnover occurs 3 times faster in rabbits compared to humans [12,15], emerging and recovery of IR damage may occur at different times. We hypothesized that a minimum period of 8 weeks would correspond with a period of 24 weeks (6 months) in humans and therefore would be sufficient to detect late IR injury in the microvasculature. However, the effects of RT might have manifested in a milder form in the bone as damaged cells would be replaced by osteoprogenitor cells in a higher turnover rate [15]. It may have been advantageous to prolong the observation period of post-IR beyond 11 weeks to determine if there may be a delayed response in the microcirculation corresponding to late IR injury as observed in human HN cancer patients.

Several points need to be considered in view of this pilot investigation. First, the sample size per group was very small and may not be sufficient to validate and ascertain adequate detection of late IR injury. Second, compared to other animal studies, 11 weeks is relatively short and might therefore have been too soon to detect vascular changes characteristic of the onset of late IR injury. Moreover, it is difficult to scale IR doses adequately for rabbits and perhaps a higher dose may have been required to achieve clinically comparable tissue pathophysiology associated with mandibular IR injury. Finally, one drawback associated with SDFI in the scope of this study is that only a small area of approximately 1 mm^2^ can be captured at a time. Since no larger mucosal surface area could be imaged, pathology could potentially be missed.

## 5. Conclusion

The present pilot study described for the first time a model in which *in vivo* oral microcirculatory response after HN IR was monitored and measured prospectively. Within the scope of this study, no significant lasting effect in terms of PVD or clinical late IR damage could be detected in the different groups after 11 weeks. In general, acute changes in vessel density and PLT counts were observed at 5 weeks after initiating IR in all groups. Marked changes in architecture (telangiectasia) and histology (loss of periosteum and vasculature) were observed after a single dose of 30 Gy. Future studies should prolong the observation period and examine injury dose-response prevalence by correlating the microcirculation, telangiectasia, and histopathology findings with the progression of IR side effects.
